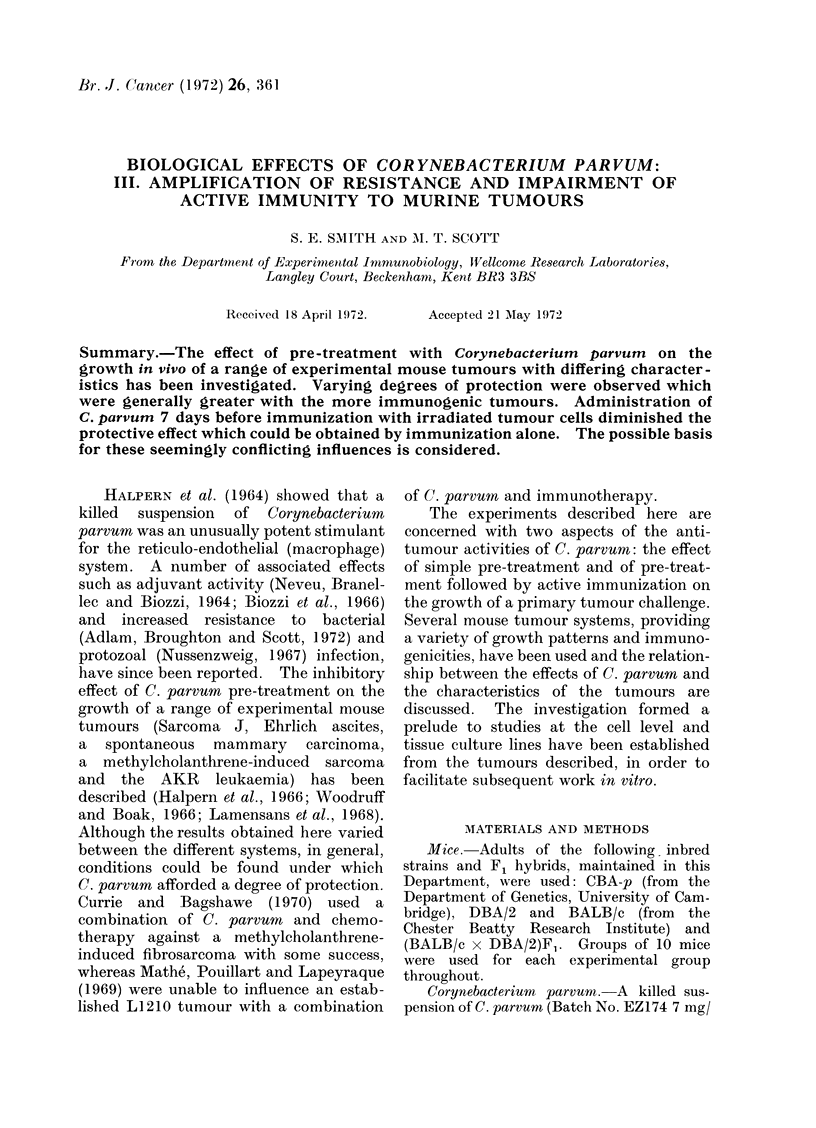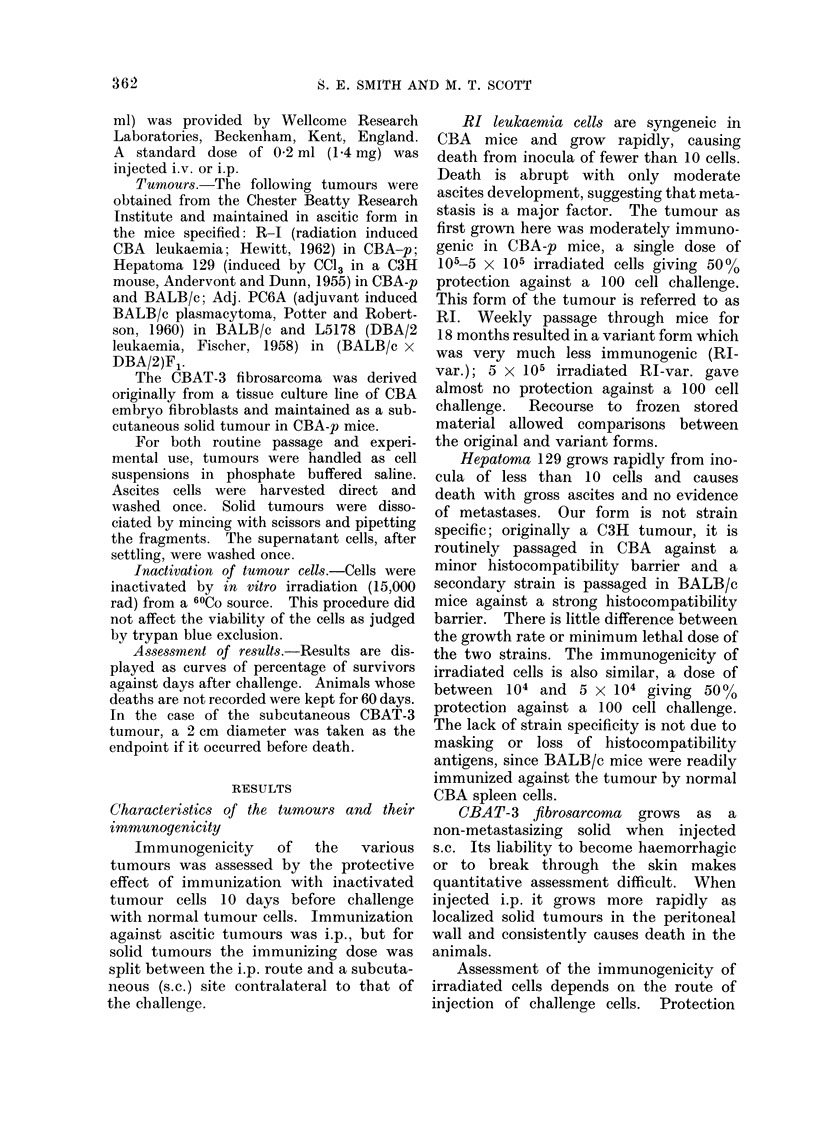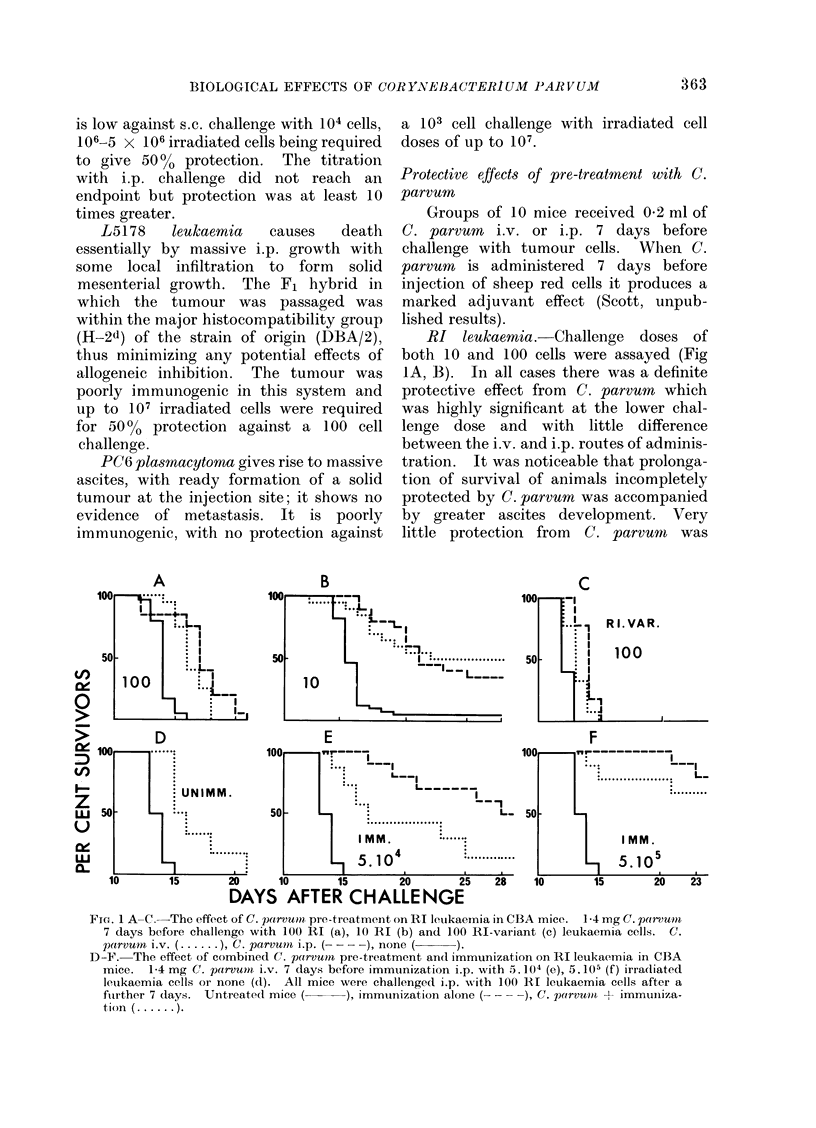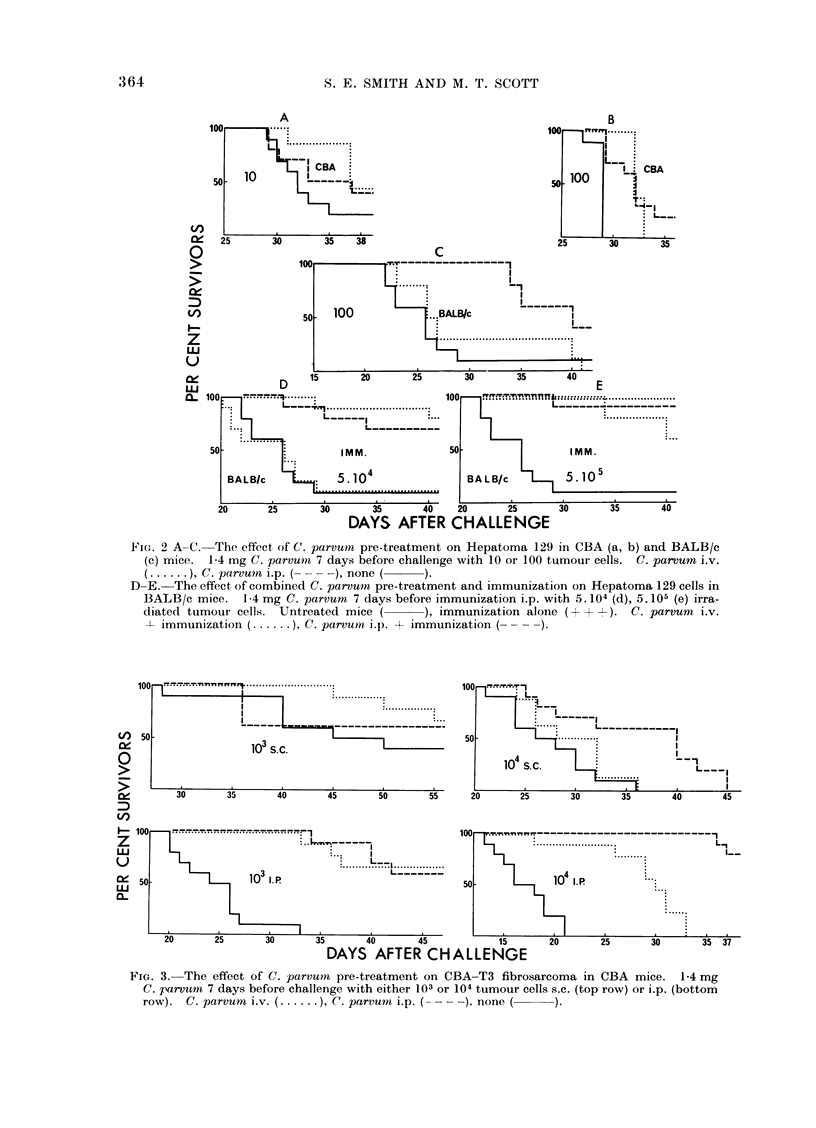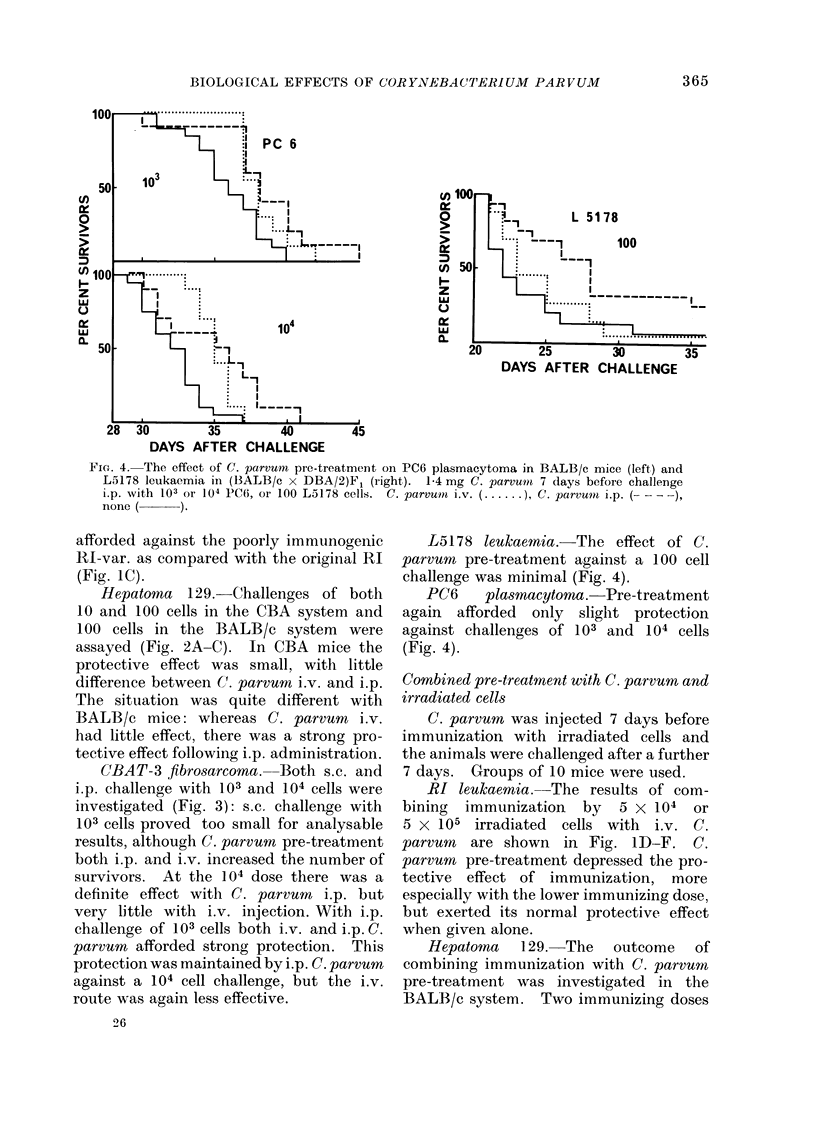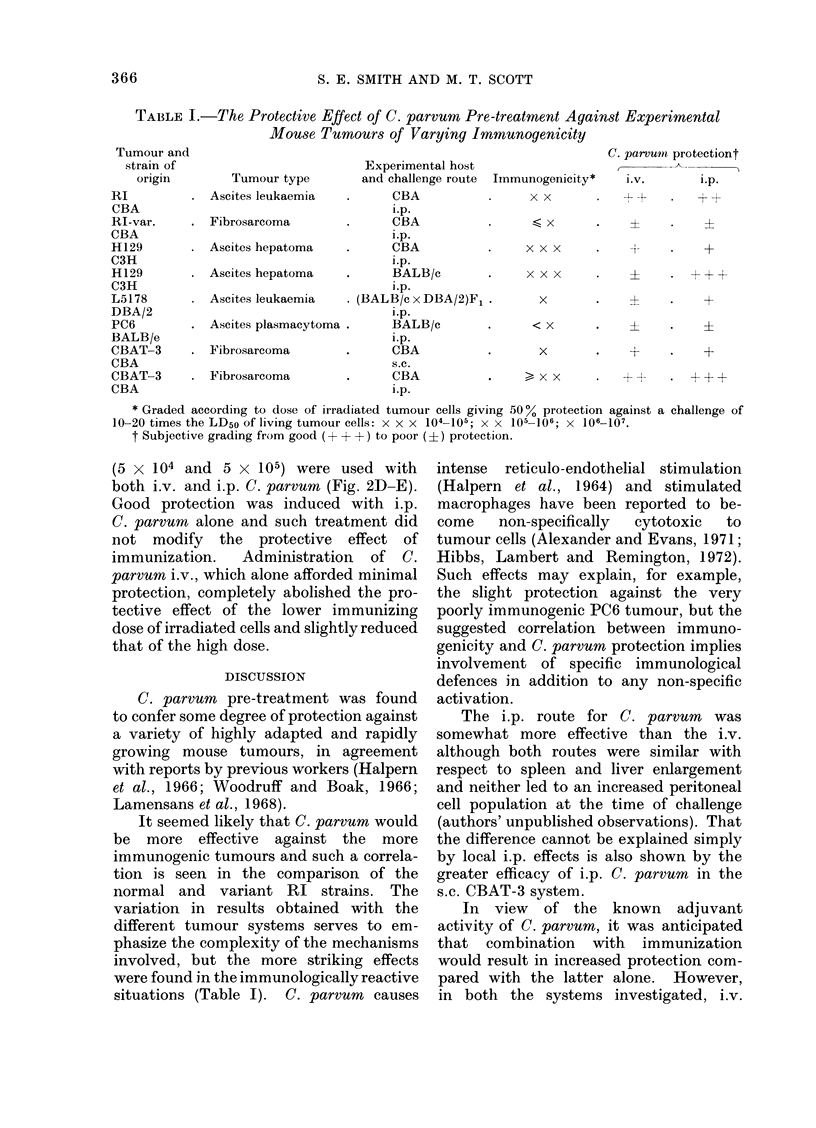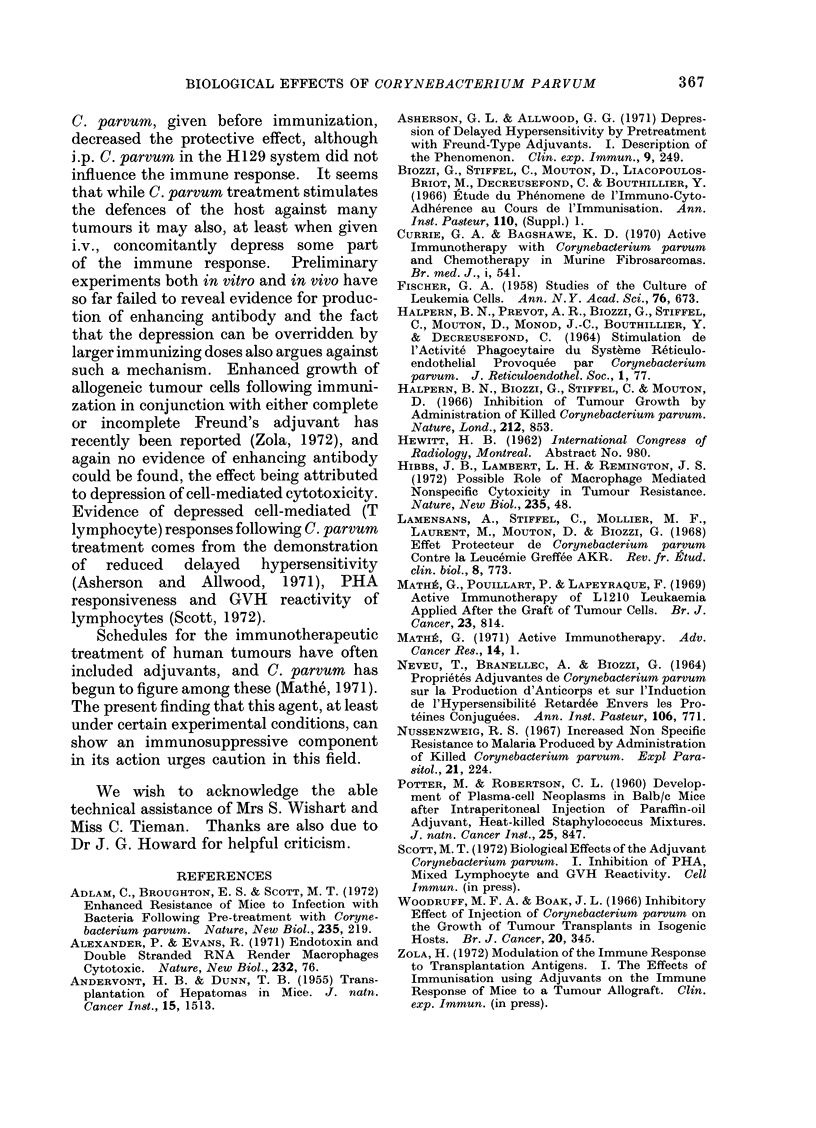# Biological Effects of Corynebacterium Parvum. III. amplification of resistance and impairment of Active Immunity to Murine Tumours

**DOI:** 10.1038/bjc.1972.47

**Published:** 1972-10

**Authors:** S. E. Smith, M. T. Scott

## Abstract

The effect of pre-treatment with *Corynebacterium parvum* on the growth *in vivo* of a range of experimental mouse tumours with differing characteristics has been investigated. Varying degrees of protection were observed which were generally greater with the more immunogenic tumours. Administration of *C. parvum* 7 days before immunization with irradiated tumour cells diminished the protective effect which could be obtained by immunization alone. The possible basis for these seemingly conflicting influences is considered.


*Br. J. Cancer* (1972) **26**, 361

### BIOLOGICAL EFFECTS OF *CORYNEBACTERIUM PARVUM*: III. AMPLIFICATION OF RESISTANCE AND IMPAIRMENT OF ACTIVE IMMUNITY TO MURINE TUMOURS

S. E. SMITH AND M. T. SCOTT

*From the Department of Experimental Immunobiology, Wellcome Research Laboratories, Langley Court, Beckenham, Kent BR3 3BS*

Received 18 April 1972. Accepted 21 May 1972

**Summary.—The effect of pre-treatment with *Corynebacterium parvum* on the growth *in vivo* of a range of experimental mouse tumours with differing characteristics has been investigated. Varying degrees of protection were observed which were generally greater with the more immunogenic tumours. Administration of *C. parvum* 7 days before immunization with irradiated tumour cells diminished the protective effect which could be obtained by immunization alone. The possible basis for these seemingly conflicting influences is considered.**

HALPERN *et al.* (1964) showed that a killed suspension of *Corynebacterium parvum* was an unusually potent stimulant for the reticulo-endothelial (macrophage) system. A number of associated effects such as adjuvant activity (Neveu, Branellec and Biozzi, 1964; Biozzi *et al.*, 1966) and increased resistance to bacterial (Adlam, Broughton and Scott, 1972) and protozoal (Nussenzweig, 1967) infection, have since been reported. The inhibitory effect of *C. parvum* pre-treatment on the growth of a range of experimental mouse tumours (Sarcoma J, Ehrlich ascites, a spontaneous mammary carcinoma, a methylcholanthrene-induced sarcoma and the AKR leukaemia) has been described (Halpern *et al.*, 1966; Woodruff and Boak, 1966; Lamensans *et al.*, 1968). Although the results obtained here varied between the different systems, in general, conditions could be found under which *C. parvum* afforded a degree of protection. Currie and Bagshawe (1970) used a combination of *C. parvum* and chemotherapy against a methylcholanthrene-induced fibrosarcoma with some success, whereas Mathé, Pouillart and Lapeyraque (1969) were unable to influence an established L1210 tumour with a combination of *C. parvum* and immunotherapy.

The experiments described here are concerned with two aspects of the anti-tumour activities of *C. parvum*: the effect of simple pre-treatment and of pre-treatment followed by active immunization on the growth of a primary tumour challenge. Several mouse tumour systems, providing a variety of growth patterns and immunogenicities, have been used and the relationship between the effects of *C. parvum* and the characteristics of the tumours are discussed. The investigation formed a prelude to studies at the cell level and tissue culture lines have been established from the tumours described, in order to facilitate subsequent work *in vitro*.

### MATERIALS AND METHODS

*Mice.*—Adults of the following inbred strains and $F_1$ hybrids, maintained in this Department, were used: CBA-*p* (from the Department of Genetics, University of Cambridge), DBA/2 and BALB/c (from the Chester Beatty Research Institute) and (BALB/c × DBA/2)$F_1$. Groups of 10 mice were used for each experimental group throughout.

*Corynebacterium parvum.*—A killed suspension of *C. parvum* (Batch No. EZ174 7 mg/

ml) was provided by Wellcome Research Laboratories, Beckenham, Kent, England. A standard dose of 0·2 ml (1·4 mg) was injected i.v. or i.p.

*Tumours.*—The following tumours were obtained from the Chester Beatty Research Institute and maintained in ascitic form in the mice specified: R–I (radiation induced CBA leukaemia; Hewitt, 1962) in CBA-*p*; Hepatoma 129 (induced by $CCl_3$ in a C3H mouse, Andervont and Dunn, 1955) in CBA-*p* and BALB/c; Adj. PC6A (adjuvant induced BALB/c plasmacytoma, Potter and Robertson, 1960) in BALB/c and L5178 (DBA/2 leukaemia, Fischer, 1958) in (BALB/c $\times$ DBA/2)$F_1$.

The CBAT-3 fibrosarcoma was derived originally from a tissue culture line of CBA embryo fibroblasts and maintained as a subcutaneous solid tumour in CBA-*p* mice.

For both routine passage and experimental use, tumours were handled as cell suspensions in phosphate buffered saline. Ascites cells were harvested direct and washed once. Solid tumours were dissociated by mincing with scissors and pipetting the fragments. The supernatant cells, after settling, were washed once.

*Inactivation of tumour cells.*—Cells were inactivated by *in vitro* irradiation (15,000 rad) from a $^{60}Co$ source. This procedure did not affect the viability of the cells as judged by trypan blue exclusion.

*Assessment of results.*—Results are displayed as curves of percentage of survivors against days after challenge. Animals whose deaths are not recorded were kept for 60 days. In the case of the subcutaneous CBAT-3 tumour, a 2 cm diameter was taken as the endpoint if it occurred before death.

### RESULTS

### *Characteristics of the tumours and their immunogenicity*

Immunogenicity of the various tumours was assessed by the protective effect of immunization with inactivated tumour cells 10 days before challenge with normal tumour cells. Immunization against ascitic tumours was i.p., but for solid tumours the immunizing dose was split between the i.p. route and a subcutaneous (s.c.) site contralateral to that of the challenge.

*RI leukaemia cells* are syngeneic in CBA mice and grow rapidly, causing death from inocula of fewer than 10 cells. Death is abrupt with only moderate ascites development, suggesting that metastasis is a major factor. The tumour as first grown here was moderately immunogenic in CBA-*p* mice, a single dose of $10^5$–$5 \times 10^5$ irradiated cells giving 50% protection against a 100 cell challenge. This form of the tumour is referred to as RI. Weekly passage through mice for 18 months resulted in a variant form which was very much less immunogenic (RI-var.); $5 \times 10^5$ irradiated RI-var. gave almost no protection against a 100 cell challenge. Recourse to frozen stored material allowed comparisons between the original and variant forms.

*Hepatoma* 129 grows rapidly from inocula of less than 10 cells and causes death with gross ascites and no evidence of metastases. Our form is not strain specific; originally a C3H tumour, it is routinely passaged in CBA against a minor histocompatibility barrier and a secondary strain is passaged in BALB/c mice against a strong histocompatibility barrier. There is little difference between the growth rate or minimum lethal dose of the two strains. The immunogenicity of irradiated cells is also similar, a dose of between $10^4$ and $5 \times 10^4$ giving 50% protection against a 100 cell challenge. The lack of strain specificity is not due to masking or loss of histocompatibility antigens, since BALB/c mice were readily immunized against the tumour by normal CBA spleen cells.

*CBAT*-3 *fibrosarcoma* grows as a non-metastasizing solid when injected s.c. Its liability to become haemorrhagic or to break through the skin makes quantitative assessment difficult. When injected i.p. it grows more rapidly as localized solid tumours in the peritoneal wall and consistently causes death in the animals.

Assessment of the immunogenicity of irradiated cells depends on the route of injection of challenge cells. Protection is low against s.c. challenge with $10^4$ cells, $10^6$–$5 \times 10^6$ irradiated cells being required to give 50% protection. The titration with i.p. challenge did not reach an endpoint but protection was at least 10 times greater.

*L5178 leukaemia* causes death essentially by massive i.p. growth with some local infiltration to form solid mesenterial growth. The $F_1$ hybrid in which the tumour was passaged was within the major histocompatibility group (H–$2^d$) of the strain of origin (DBA/2), thus minimizing any potential effects of allogeneic inhibition. The tumour was poorly immunogenic in this system and up to $10^7$ irradiated cells were required for 50% protection against a 100 cell challenge.

*PC6 plasmacytoma* gives rise to massive ascites, with ready formation of a solid tumour at the injection site; it shows no evidence of metastasis. It is poorly immunogenic, with no protection against a $10^3$ cell challenge with irradiated cell doses of up to $10^7$.

*Protective effects of pre-treatment with C. parvum*

Groups of 10 mice received 0·2 ml of *C. parvum* i.v. or i.p. 7 days before challenge with tumour cells. When *C. parvum* is administered 7 days before injection of sheep red cells it produces a marked adjuvant effect (Scott, unpublished results).

*RI leukaemia.*—Challenge doses of both 10 and 100 cells were assayed (Fig 1A, B). In all cases there was a definite protective effect from *C. parvum* which was highly significant at the lower challenge dose and with little difference between the i.v. and i.p. routes of administration. It was noticeable that prolongation of survival of animals incompletely protected by *C. parvum* was accompanied by greater ascites development. Very little protection from *C. parvum* was

FIG. 1 A–C.—The effect of *C. parvum* pre-treatment on RI leukaemia in CBA mice. 1·4 mg *C. parvum* 7 days before challenge with 100 RI (a), 10 RI (b) and 100 RI-variant (c) leukaemia cells. *C. parvum* i.v. (......), *C. parvum* i.p. (– – – –), none (———).

D–F.—The effect of combined *C. parvum* pre-treatment and immunization on RI leukaemia in CBA mice. 1·4 mg *C. parvum* i.v. 7 days before immunization i.p. with $5.10^4$ (e), $5.10^5$ (f) irradiated leukaemia cells or none (d). All mice were challenged i.p. with 100 RI leukaemia cells after a further 7 days. Untreated mice (———), immunization alone (– – – –), *C. parvum* + immunization (......).

FIG. 2 A–C.—The effect of *C. parvum* pre-treatment on Hepatoma 129 in CBA (a, b) and BALB/c (c) mice. 1·4 mg *C. parvum* 7 days before challenge with 10 or 100 tumour cells. *C. parvum* i.v. (. . . . . .), *C. parvum* i.p. (– – – –), none (————).

D–E.—The effect of combined *C. parvum* pre-treatment and immunization on Hepatoma 129 cells in BALB/c mice. 1·4 mg *C. parvum* 7 days before immunization i.p. with $5.10^4$ (d), $5.10^5$ (e) irradiated tumour cells. Untreated mice (————), immunization alone (+ + + +). *C. parvum* i.v. + immunization (. . . . . .), *C. parvum* i.p. + immunization (– – – –).

FIG. 3.—The effect of *C. parvum* pre-treatment on CBA–T3 fibrosarcoma in CBA mice. 1·4 mg *C. parvum* 7 days before challenge with either $10^3$ or $10^4$ tumour cells s.c. (top row) or i.p. (bottom row). *C. parvum* i.v. (. . . . . .), *C. parvum* i.p. (– – – –). none (————).

FIG. 4.—The effect of *C. parvum* pre-treatment on PC6 plasmacytoma in BALB/c mice (left) and L5178 leukaemia in (BALB/c × DBA/2)$F_1$ (right). 1·4 mg *C. parvum* 7 days before challenge i.p. with $10^3$ or $10^4$ PC6, or 100 L5178 cells. *C. parvum* i.v. (......), *C. parvum* i.p. (- - - -), none (————).

afforded against the poorly immunogenic RI-var. as compared with the original RI (Fig. 1C).

*Hepatoma* 129.—Challenges of both 10 and 100 cells in the CBA system and 100 cells in the BALB/c system were assayed (Fig. 2A–C). In CBA mice the protective effect was small, with little difference between *C. parvum* i.v. and i.p. The situation was quite different with BALB/c mice: whereas *C. parvum* i.v. had little effect, there was a strong protective effect following i.p. administration.

*CBAT*-3 *fibrosarcoma*.—Both s.c. and i.p. challenge with $10^3$ and $10^4$ cells were investigated (Fig. 3): s.c. challenge with $10^3$ cells proved too small for analysable results, although *C. parvum* pre-treatment both i.p. and i.v. increased the number of survivors. At the $10^4$ dose there was a definite effect with *C. parvum* i.p. but very little with i.v. injection. With i.p. challenge of $10^3$ cells both i.v. and i.p. *C. parvum* afforded strong protection. This protection was maintained by i.p. *C. parvum* against a $10^4$ cell challenge, but the i.v. route was again less effective.

*L*5178 *leukaemia*.—The effect of *C. parvum* pre-treatment against a 100 cell challenge was minimal (Fig. 4).

*PC*6 *plasmacytoma*.—Pre-treatment again afforded only slight protection against challenges of $10^3$ and $10^4$ cells (Fig. 4).

*Combined pre-treatment with C. parvum and irradiated cells*

*C. parvum* was injected 7 days before immunization with irradiated cells and the animals were challenged after a further 7 days. Groups of 10 mice were used.

*RI leukaemia*.—The results of combining immunization by $5 \times 10^4$ or $5 \times 10^5$ irradiated cells with i.v. *C. parvum* are shown in Fig. 1D–F. *C. parvum* pre-treatment depressed the protective effect of immunization, more especially with the lower immunizing dose, but exerted its normal protective effect when given alone.

*Hepatoma* 129.—The outcome of combining immunization with *C. parvum* pre-treatment was investigated in the BALB/c system. Two immunizing doses

TABLE I.—*The Protective Effect of C. parvum Pre-treatment Against Experimental Mouse Tumours of Varying Immunogenicity*

| Tumour and strain of origin | Tumour type | Experimental host and challenge route | Immunogenicity* | *C. parvum* protection† | |
|---|---|---|---|---|---|
| | | | | i.v. | i.p. |
| RI CBA | Ascites leukaemia | CBA i.p. | × × | + + | + + |
| RI-var. CBA | Fibrosarcoma | CBA i.p. | ⩽ × | ± | ± |
| H129 C3H | Ascites hepatoma | CBA i.p. | × × × | + | + |
| H129 C3H | Ascites hepatoma | BALB/c i.p. | × × × | ± | + + + |
| L5178 DBA/2 | Ascites leukaemia | (BALB/c × DBA/2)$F_1$ i.p. | × | ± | + |
| PC6 BALB/c | Ascites plasmacytoma | BALB/c i.p. | < × | ± | ± |
| CBAT-3 CBA | Fibrosarcoma | CBA s.c. | × | + | + |
| CBAT-3 CBA | Fibrosarcoma | CBA i.p. | ⩾ × × | + + | + + + |

* Graded according to dose of irradiated tumour cells giving 50% protection against a challenge of 10–20 times the $LD_{50}$ of living tumour cells: × × × $10^4$–$10^5$; × × $10^5$–$10^6$; × $10^6$–$10^7$.

† Subjective grading from good (+ + +) to poor (±) protection.

($5 \times 10^4$ and $5 \times 10^5$) were used with both i.v. and i.p. *C. parvum* (Fig. 2D–E). Good protection was induced with i.p. *C. parvum* alone and such treatment did not modify the protective effect of immunization. Administration of *C. parvum* i.v., which alone afforded minimal protection, completely abolished the protective effect of the lower immunizing dose of irradiated cells and slightly reduced that of the high dose.

### DISCUSSION

*C. parvum* pre-treatment was found to confer some degree of protection against a variety of highly adapted and rapidly growing mouse tumours, in agreement with reports by previous workers (Halpern *et al.*, 1966; Woodruff and Boak, 1966; Lamensans *et al.*, 1968).

It seemed likely that *C. parvum* would be more effective against the more immunogenic tumours and such a correlation is seen in the comparison of the normal and variant RI strains. The variation in results obtained with the different tumour systems serves to emphasize the complexity of the mechanisms involved, but the more striking effects were found in the immunologically reactive situations (Table I). *C. parvum* causes intense reticulo-endothelial stimulation (Halpern *et al.*, 1964) and stimulated macrophages have been reported to become non-specifically cytotoxic to tumour cells (Alexander and Evans, 1971; Hibbs, Lambert and Remington, 1972). Such effects may explain, for example, the slight protection against the very poorly immunogenic PC6 tumour, but the suggested correlation between immunogenicity and *C. parvum* protection implies involvement of specific immunological defences in addition to any non-specific activation.

The i.p. route for *C. parvum* was somewhat more effective than the i.v. although both routes were similar with respect to spleen and liver enlargement and neither led to an increased peritoneal cell population at the time of challenge (authors' unpublished observations). That the difference cannot be explained simply by local i.p. effects is also shown by the greater efficacy of i.p. *C. parvum* in the s.c. CBAT-3 system.

In view of the known adjuvant activity of *C. parvum*, it was anticipated that combination with immunization would result in increased protection compared with the latter alone. However, in both the systems investigated, i.v.

*C. parvum*, given before immunization, decreased the protective effect, although i.p. *C. parvum* in the H129 system did not influence the immune response. It seems that while *C. parvum* treatment stimulates the defences of the host against many tumours it may also, at least when given i.v., concomitantly depress some part of the immune response. Preliminary experiments both *in vitro* and *in vivo* have so far failed to reveal evidence for production of enhancing antibody and the fact that the depression can be overridden by larger immunizing doses also argues against such a mechanism. Enhanced growth of allogeneic tumour cells following immunization in conjunction with either complete or incomplete Freund's adjuvant has recently been reported (Zola, 1972), and again no evidence of enhancing antibody could be found, the effect being attributed to depression of cell-mediated cytotoxicity. Evidence of depressed cell-mediated (T lymphocyte) responses following *C. parvum* treatment comes from the demonstration of reduced delayed hypersensitivity (Asherson and Allwood, 1971), PHA responsiveness and GVH reactivity of lymphocytes (Scott, 1972).

Schedules for the immunotherapeutic treatment of human tumours have often included adjuvants, and *C. parvum* has begun to figure among these (Mathé, 1971). The present finding that this agent, at least under certain experimental conditions, can show an immunosuppressive component in its action urges caution in this field.

We wish to acknowledge the able technical assistance of Mrs S. Wishart and Miss C. Tieman. Thanks are also due to Dr J. G. Howard for helpful criticism.

### REFERENCES

ADLAM, C., BROUGHTON, E. S. & SCOTT, M. T. (1972) Enhanced Resistance of Mice to Infection with Bacteria Following Pre-treatment with *Corynebacterium parvum*. *Nature, New Biol.*, **235**, 219.

ALEXANDER, P. & EVANS, R. (1971) Endotoxin and Double Stranded RNA Render Macrophages Cytotoxic. *Nature, New Biol.*, **232**, 76.

ANDERVONT, H. B. & DUNN, T. B. (1955) Transplantation of Hepatomas in Mice. *J. natn. Cancer Inst.*, **15**, 1513.

ASHERSON, G. L. & ALLWOOD, G. G. (1971) Depression of Delayed Hypersensitivity by Pretreatment with Freund-Type Adjuvants. I. Description of the Phenomenon. *Clin. exp. Immun.*, **9**, 249.

BIOZZI, G., STIFFEL, C., MOUTON, D., LIACOPOULOS-BRIOT, M., DECREUSEFOND, C. & BOUTHILLIER, Y. (1966) Étude du Phénomene de l'Immuno-Cyto-Adhérence au Cours de l'Immunisation. *Ann. Inst. Pasteur*, **110**, (Suppl.) 1.

CURRIE, G. A. & BAGSHAWE, K. D. (1970) Active Immunotherapy with *Corynebacterium parvum* and Chemotherapy in Murine Fibrosarcomas. *Br. med. J.*, i, 541.

FISCHER, G. A. (1958) Studies of the Culture of Leukemia Cells. *Ann. N.Y. Acad. Sci.*, **76**, 673.

HALPERN, B. N., PREVOT, A. R., BIOZZI, G., STIFFEL, C., MOUTON, D., MONOD, J.-C., BOUTHILLIER, Y. & DECREUSEFOND, C. (1964) Stimulation de l'Activité Phagocytaire du Système Réticulo-endothelial Provoquée par *Corynebacterium parvum*. *J. Reticuloendothel. Soc.*, **1**, 77.

HALPERN, B. N., BIOZZI, G., STIFFEL, C. & MOUTON, D. (1966) Inhibition of Tumour Growth by Administration of Killed *Corynebacterium parvum*. *Nature, Lond.*, **212**, 853.

HEWITT, H. B. (1962) *International Congress of Radiology, Montreal.* Abstract No. 980.

HIBBS, J. B., LAMBERT, L. H. & REMINGTON, J. S. (1972) Possible Role of Macrophage Mediated Nonspecific Cytoxicity in Tumour Resistance. *Nature, New Biol.*, **235**, 48.

LAMENSANS, A., STIFFEL, C., MOLLIER, M. F., LAURENT, M., MOUTON, D. & BIOZZI, G. (1968) Effet Protecteur de *Corynebacterium parvum* Contre la Leucémie Greffée AKR. *Rev. fr. Étud. clin. biol.*, **8**, 773.

MATHÉ, G., POUILLART, P. & LAPEYRAQUE, F. (1969) Active Immunotherapy of L1210 Leukaemia Applied After the Graft of Tumour Cells. *Br. J. Cancer*, **23**, 814.

MATHÉ, G. (1971) Active Immunotherapy. *Adv. Cancer Res.*, **14**, 1.

NEVEU, T., BRANELLEC, A. & BIOZZI, G. (1964) Propriétés Adjuvantes de *Corynebacterium parvum* sur la Production d'Anticorps et sur l'Induction de l'Hypersensibilité Retardée Envers les Protéines Conjuguées. *Ann. Inst. Pasteur*, **106**, 771.

NUSSENZWEIG, R. S. (1967) Increased Non Specific Resistance to Malaria Produced by Administration of Killed *Corynebacterium parvum*. *Expl Parasitol.*, **21**, 224.

POTTER, M. & ROBERTSON, C. L. (1960) Development of Plasma-cell Neoplasms in Balb/c Mice after Intraperitoneal Injection of Paraffin-oil Adjuvant, Heat-killed Staphylococcus Mixtures. *J. natn. Cancer Inst.*, **25**, 847.

SCOTT, M. T. (1972) Biological Effects of the Adjuvant *Corynebacterium parvum*. I. Inhibition of PHA, Mixed Lymphocyte and GVH Reactivity. *Cell Immun.* (in press).

WOODRUFF, M. F. A. & BOAK, J. L. (1966) Inhibitory Effect of Injection of *Corynebacterium parvum* on the Growth of Tumour Transplants in Isogenic Hosts. *Br. J. Cancer*, **20**, 345.

ZOLA, H. (1972) Modulation of the Immune Response to Transplantation Antigens. I. The Effects of Immunisation using Adjuvants on the Immune Response of Mice to a Tumour Allograft. *Clin. exp. Immun.* (in press).